# Anatomic evaluation of the insertional footprints of the iliofemoral and ischiofemoral ligaments: a cadaveric study

**DOI:** 10.1186/s12891-020-03848-4

**Published:** 2020-12-09

**Authors:** Yasuaki Tamaki, Tomohiro Goto, Keizo Wada, Daisuke Hamada, Yoshihiro Tsuruo, Koichi Sairyo

**Affiliations:** 1 Department of Orthopedics, Institute of Biomedical Sciences, Tokushima University Graduate School, 3-18-15 Kuramoto, Tokushima, 770-8503 Japan; 2 Department of Anatomy and Cell Biology, Institute of Biomedical Sciences, Tokushima University Graduate School, 3-18-15 Kuramoto, Tokushima, 770-8503 Japan

**Keywords:** Hip joint, Capsular ligament, Total hip arthroplasty, Minimally invasive surgery, Iliofemoral ligament, Ischiofemoral ligament

## Abstract

**Background:**

An understanding of the insertional footprints of the capsular ligaments of the hip is important for preserving hip function and stability given the increasing number of minimally invasive hip surgeries being performed under a limited surgical view. However, it is difficult to detect these ligaments intraoperatively and many surgeons may not fully appreciate their complex anatomy. The aims of this study were to quantify the proximal and distal footprints of the iliofemoral ligament (ILFL) and ischiofemoral ligament (ISFL) and to estimate the location of the corresponding osseous landmarks on the proximal femur, which can be detected easily during surgery.

**Methods:**

Twelve hip joints from Japanese fresh frozen cadavers were used. All muscle, fascia, nerve tissue, and vessels were removed to expose the intact capsular ligaments of the hip. The length and width of the proximal and distal footprints of the ILFL and ISFL were measured and their relationship to osseous structures was evaluated, including the intertrochanteric line, femoral neck, and lesser trochanter.

**Results:**

The mean length of the distal medial arm of the ILFL footprint was 17.9 mm and the mean width was 9.0 mm. The mean length of the distal lateral arm of the ILFL footprint was 23.0 mm and the mean width was 9.7 mm. For the footprint of the medial arm, the insertion was in the distal third of the intertrochanteric line and that of the lateral arm was in the proximal 42% of this line. The mean distance from the lesser trochanter to the footprint of the medial arm was 24.6 mm. The mean length of the distal ISFL footprint was 11.3 mm and the mean width was 6.9 mm. The footprint of the distal ISFL was located forward of the femoral neck axis in all specimens.

**Conclusions:**

Understanding the size and location of each capsular ligament footprint in relation to an osseous landmark may help surgeons to manage the hip capsule intraoperatively even under a narrow surgical view. The findings of this study underscore the importance of recognizing that the distal ISFL footprint is located relatively forward and very close to the distal lateral arm footprint.

## Background

The hip capsule includes three main ligaments, namely, the iliofemoral ligament (ILFL), ischiofemoral ligament (ISFL), and pubofemoral ligament, which form a lining surrounding the hip joint and are thought to stabilize the joint [1–6]. Preservation or reconstruction of these capsular ligaments is important in modern hip joint surgeries, such as arthroplasty, arthroscopy, and open surgical procedures [7–11]. In particular, minimally invasive surgery has become a focus in total hip arthroplasty (THA), and preservation of the hip capsule is an important consideration [9–11]. However, iatrogenic instability of the hip due to capsulotomy or poor reconstruction of the capsule during hip arthroscopy remains a problem [7, 12] and leads to progression of osteoarthritis in the early postoperative period. Most surgeons are aware of the anatomy of the capsular ligaments; however, these structures may be difficult to identify precisely in the actual surgical field because the surgical view is very limited, potentially giving rise to uncertainty during ligament preservation or reconstruction procedures in the clinical setting.

The anatomical features of the capsular ligaments of the hip are well described in the literature. The ILFL originates proximally from the inferior portion of the anterior inferior iliac spine (AIIS) and distally divides into a medial arm (MA) and a lateral arm (LA). These ligaments insert distally along the intertrochanteric line of the femur with a thinner central portion between them [13–16]. According to the latest anatomical knowledge on the ILFL, the inferior edge of the AIIS, which is the proximal attachment site of the ILFL, is characterized by an osseous impression, a broad attachment width, and distributed fibrocartilage [15]. Furthermore, the gluteus minimus tendon is connected to the distal part of the LA, the deep aponeurosis of the iliopsoas is connected to the distal part of the MA, and each capsular complex inserts to the proximal and distal portions of the intertrochanteric line [16]. These insights, which show that the capsular ligaments form a complex with muscles around the hip, underscore the importance of the capsular ligaments as joint stabilizers and the need for more practical information, including the size and proportion of each footprint and its location relative to the intertrochanteric line, in order to prevent capsular damage intraoperatively. There have been relatively few anatomical studies of the ISFL. The ISFL originates near the root of the ischial ramus and inserts distally at the base of the greater trochanter. However, it is unclear whether the distal footprint is located at the superior aspect of the femoral neck or slightly anterior to the axis of the femoral neck [13, 14].

The literature contains many descriptions of the clinical benefits of preserving the hip capsule [3–6], including some reports suggesting that preservation of the capsule could have benefits for patients by preserving the defences of the native hip against hypermobility, impingement, subluxation, and edge-loading [5, 9, 11]. Surgeons performing minimally invasive THA now try to preserve and repair the capsular ligaments as much as possible and have introduced muscle-sparing strategies, including anterior and anterolateral approaches, which are gaining in popularity as methods that facilitate rapid postoperative recovery [17]. Martin et al. reported that the ILFL is the greatest inhibitor of external rotation in extension and that the ISFL restricts internal rotation in extension and flexion [4]. Because the position of the hip in external rotation during extension could be a risk factor for anterior dislocation and its position in internal rotation during flexion could increase the risk of posterior dislocation, the MA of the ILFL and ISFL should be preserved as far as possible to ensure joint stability after THA. Moreover, when using an anterior or anterolateral approach, the ISFL is thought to be a major factor restricting elevation of the femur with external rotation. Therefore, elevation of the femur during stem implantation and management of ISFL resection is important for stem alignment, including flexion and antetorsion angles. To prevent damage to the hip capsular ligaments and to reconstruct them correctly, surgeons should detect the precise anatomy of these ligaments intraoperatively. To this end, they need a detailed knowledge of the insertional footprints of the capsular ligaments, and this may be improved by awareness of certain bony landmarks that are easily recognized and palpable intraoperatively.

The aims of this study were to examine the proximal and distal footprints of the ILFL and ISFL in anatomical detail and to determine the osseous landmarks that can be detected during surgery.

## Methods

This study was approved by the authors’ institutional review board (approval number: 2068) and performed in accordance with the principles of the Declaration of Helsinki. Each donor and their family provided written informed consent to use their body for research when they donated their body to the institution. Twelve hips from Japanese fresh frozen cadavers (8 male, 4 female) were used in the study. All specimens included the entire hemipelvis and femur resected at the sacroiliac joint, the pubic tubercle, and the knee joint. Mean age at the time of death was 85.3 (range, 69–96) years. Details of the antemortem weight, height, physical activity, and cause of death were not available. None of the hips had undergone a previous surgical procedure. The osseous morphology of each specimen was evaluated by computed tomography. There was no evidence of osteoarthritic change, such as marked joint space narrowing or osteophytes. Each specimen was removed from the freezer and thawed for 48 h at 23 °C (room temperature) before the study was performed. After thawing, all muscle, fascia, nerve tissue, and vessels were removed to expose the intact capsular ligaments of the hip.

**Fig. 1:**
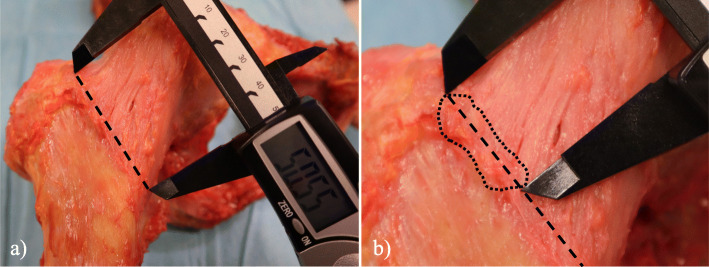
Photograph of the anterior aspect of the hip showing how the calliper was used to measure each ligament. **a** Measurement of the intertrochanteric line. **b** The length of the lateral arm of the iliofemoral ligament was measured. A black dashed line shows the intertrochanteric line. A black dotted line shows the outline of the footprint of the lateral arm

**Fig. 2:**
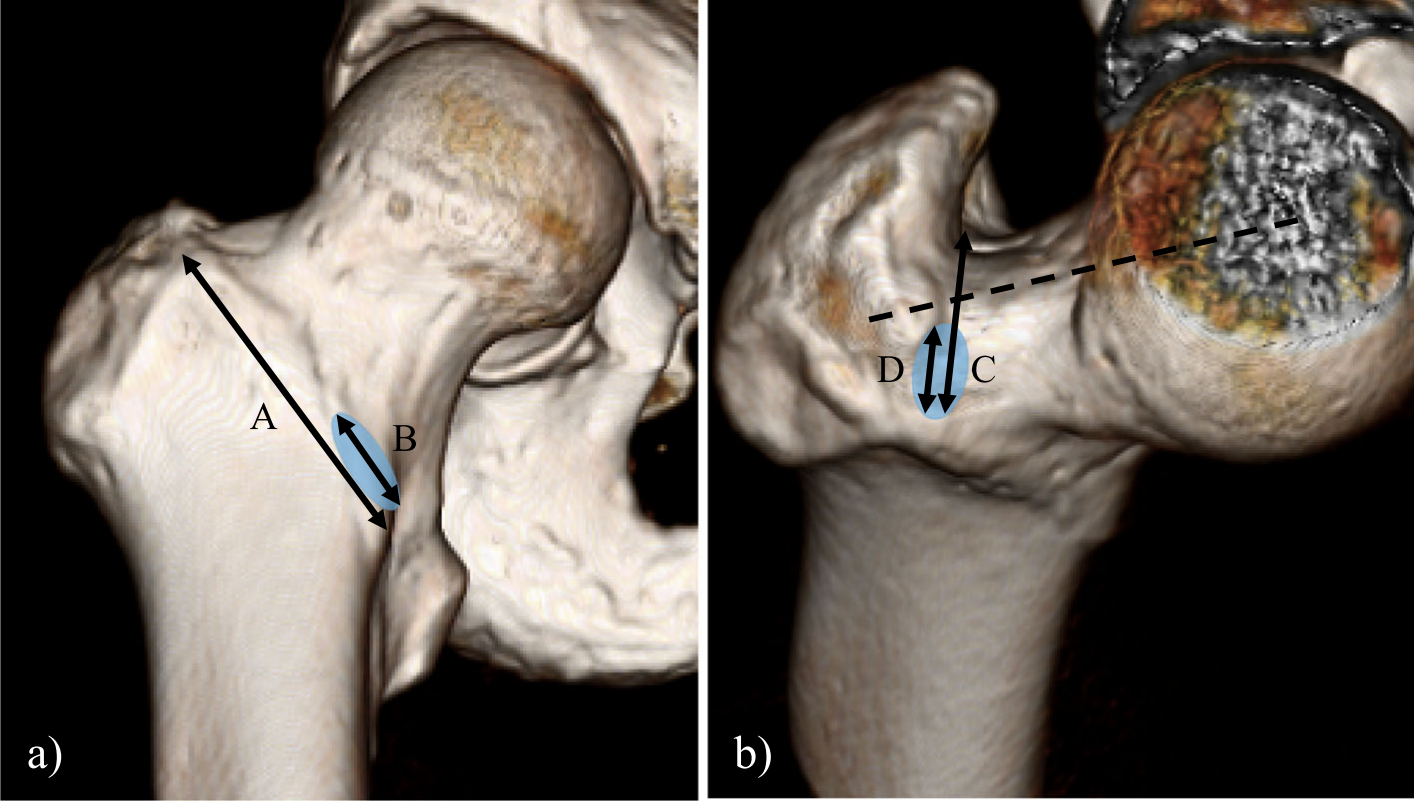
Three-dimensional bone model showing the relationship between osseous landmarks and the footprints of the capsular ligaments. **a** Anteroposterior view of the femur. Line A indicates the length of the intertrochanteric line. Line B indicates the length of the distal footprint of the medial arm. F/I ratio = B/A × 100 (%). The circle shows the footprint of the distal medial arm. **b** Superior view of the femoral neck. Line C indicates the anteroposterior length of the femoral neck at the junction of the greater trochanter and femoral neck. Line D indicates the length of the distal footprint of the ischiofemoral ligament. F/FN ratio = D/C × 100 (%). The circle shows the footprint of the distal ischiofemoral ligament. The dotted line shows the femoral neck axis

The proximal and distal MA and LA footprints of the ILFL and ISFL were obtained. When the footprint was unclear, the hip capsule was carefully incised along the outline of the ligament with a scalpel blade to detect the precise footprint in intra-capsular and extra-capsular views. Each ligament footprint was outlined with a marking pen. Next, the length and width of the footprint were measured using an electronic calliper (Fig. 1). The shape of the footprint was found to be ellipsoid and the maximum length and width were determined relative to the minimum diameter of the footprint. Also measured were the osseous structures, including the length of the intertrochanteric line, which is a ridge on the femur and located on the anterior aspect of the junction of the femoral neck and shaft, and the anteroposterior length of the femoral neck at the junction of the femoral neck and greater trochanter (Fig. 2). The anteroposterior length of the femoral neck was measured using an electronic calliper while supporting the anterior and posterior aspects of the femoral neck at its junction with the greater trochanter. All lengths were calculated as the direct distance using the electronic calliper (measurement range, 0.01–150 mm).

The relationship between the osseous structures and each footprint was evaluated. For the ILFL, we evaluated the F/I ratio, defined as the ratio of the distal footprint length to the length of the intertrochanteric line (Fig. 2a) and the distance from the tip of the lesser trochanter to the centre of the distal footprint of the MA. For the ISFL, we evaluated the F/FN ratio, defined as the ratio of the footprint length of the distal ISFL to the anteroposterior length of the femoral neck (Fig. 2b). The femoral neck axis was determined as the line between the centre of the femoral head and that of the isthmus of the neck in the axial plane. The footprint of each ligament was marked based on consensus between two observers; each measurement was performed twice and the mean value was used for analysis. The intraclass correlation coefficient (ICC) was used to evaluate the reliability of each measurement.

## Results

The capsular ligaments were detected as areas of increased thickness in the articular capsule and by confirmation of ligament fibres on direct view. The ILFL (including the MA and LA) and ISFL were clearly identified in all specimens. The intra-observer reliability of each origin and footprint measurement (length/width) was good to excellent (ICC, 0.72/0.62 for the origin of the ILFL, 0.87/0.88 for the footprint of the MA of the ILFL, 0.78/0.68 for the footprint of the LA of the ILFL, 0.67/0.79 for the origin of the ISFL, and 0.87/0.70 for the footprint of the ISFL).

### Iliofemoral ligament

**Fig. 3:**
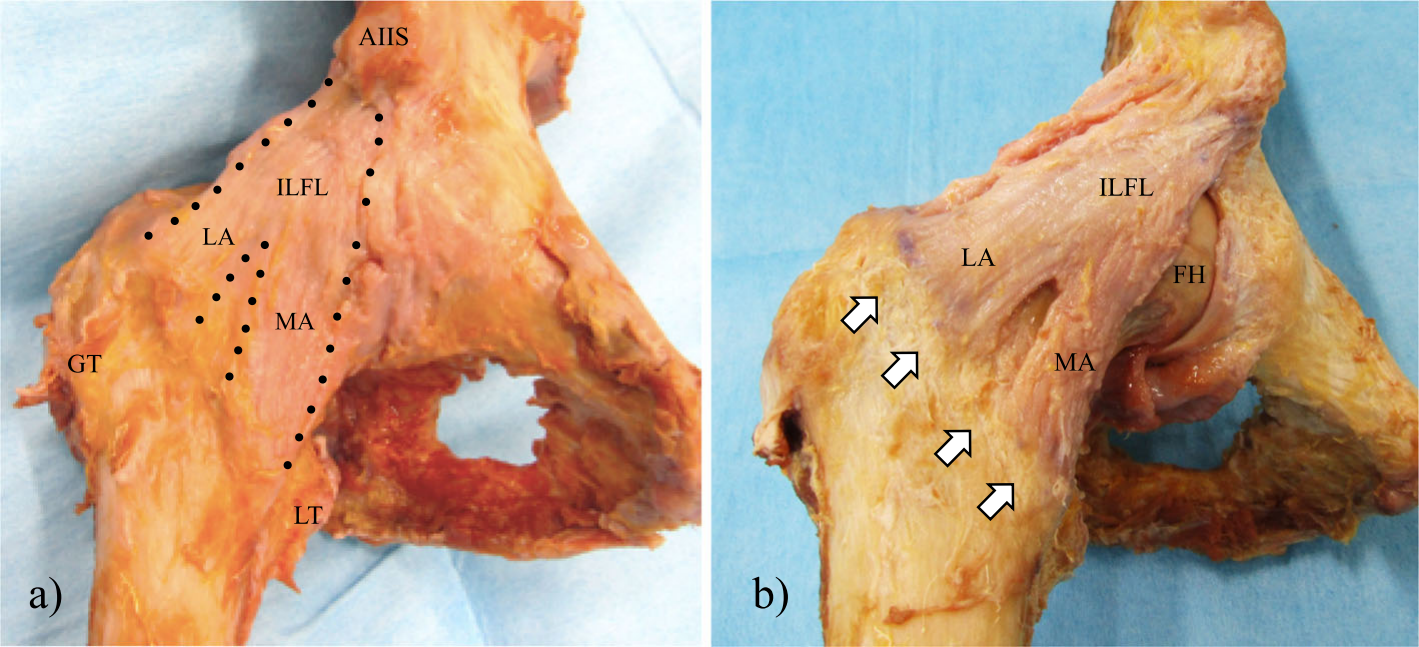
Photographs of the iliofemoral ligament (ILFL). **a** A black dotted line shows the outline of the ILFL. The ILFL consists of a lateral arm (LA) and a medial arm (MA). **b** The photograph shows the anterior hip capsule after removal of the thin fibres and the pubofemoral ligament. The ILFL and its insertions can be observed clearly. The white arrows show the intertrochanteric line, which includes the distal footprints of the LA and MA. AIIS, anterior inferior iliac spine; GT, greater trochanter; LT, lesser trochanter

The ILFL was in the anterior hip capsule. The origin of the ILFL was wrapped around the base of the AIIS. The distal portion of this ligament was split into the MA and LA (Fig. 3a). The MA ran across the anterior surface of the femoral head and inserted into the inferior portion of the intertrochanteric line, which was located in front of the lesser trochanter (Fig. 3b). The distal footprint of the MA formed a bony prominence that was detectable in all cases. The mean length of the distal MA footprint was 17.9 mm and the mean width was 9.0 mm (Table 1). The mean F/I ratio of the MA was 32.9%. The mean distance from the tip of the lesser trochanter to the distal footprint of the MA was 24.6 mm (Table 2). The LA was located at the superolateral surface of the femoral head and attached along the superior aspect of the intertrochanteric line (Fig. 3b). The mean length of the distal LA footprint was 23.0 mm and the mean width was 9.7 mm (Table 1). The mean F/I ratio of the LA was 42.0%. The capsule at the gap between the footprint of the distal MA and distal LA was very thin; the mean distance of this gap was 11.7 mm (range, 8.7–14.7 mm; standard deviation [SD] 1.8).

**Table 1:** Measurement of the footprints of the iliofemoral and ischiofemoral ligaments

	Length (mm)			Width (mm)		
	Mean	Range	SD	Mean	Range	SD
Proximal ILFL	18.3	15.5–21.5	1.9	8.1	6.7–9.7	1.0
Distal MA of the ILFL	17.9	14.9–20.2	1.6	9.0	7.8–11.4	1.1
Distal LA of the ILFL	23.0	17.3–27.8	3.5	9.7	8.2–12.0	1.1
Proximal ISFL	51.6	45.4–58.7	4.1	9.3	6.3–12.8	1.7
Distal ISFL	11.3	10.0–14.3	1.1	6.9	5.3–9.2	1.0

*ILFL* iliofemoral ligament, *ISFL* ischiofemoral ligament, *LA* lateral arm, *MA* medial arm, *SD* standard deviation

**Table 2:** Relationship between the capsular ligament of the hip and osseous landmarks

	Mean	Range	SD
Iliofemoral ligament			
Length of intertrochanteric line (mm)	54.7	45.2–61.2	4.6
F/I ratio of the MA (%)	32.9	27.2–37.6	3.4
F/I ratio of the LA (%)	42.0	31.3–48.1	4.8
Distance from LT to medial ILFL (mm)	24.6	19.9–28.5	2.9
Ischiofemoral ligament			
Femoral neck length (mm)	26.7	23.1–33.6	3.1
F/FN ratio of ISFL (%)	42.6	36.9–46.5	3.3

*F/I* footprint/intertrochanteric line, *F/FN* footprint/femoral neck width, *ILFL* iliofemoral ligament, *ISFL* ischiofemoral ligament, *LA* lateral arm, *LT* lesser trochanter, *MA* medial arm

### Ischiofemoral ligament

**Fig. 4:**
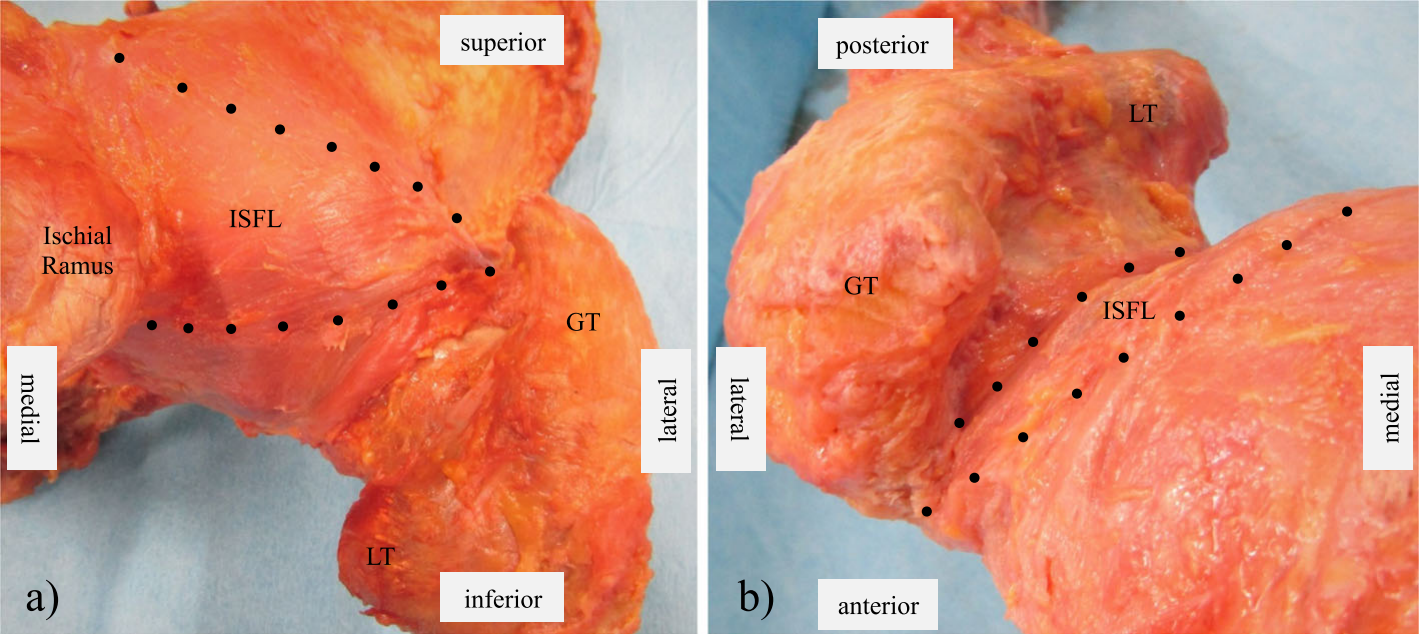
Photographs of the ischiofemoral ligament (ISFL). A black dotted line shows the outline of the ISFL. Posterior (**a**) and superior (**b**) views of the right hip joint. The femoral footprint of the ISFL is located at the junction of the femoral neck and greater trochanter in front of the femoral neck axis. GT, greater trochanter; LT, lesser trochanter

The ISFL was in the posterior hip capsule and had a triangular shape. The proximal footprint was broadly attached to the ischial circumference of the acetabular rim, where the thickest fibres reinforcing the posterior hip capsule were attached at the base of the ischial ramus (Fig. 4a). The ISFL ran obliquely along the posterior aspect of the femoral head and inserted at its junction with the greater trochanter. In all cases, the distal ISFL footprint was located anterior to the femoral neck axis (Fig. 4b). The distal ISFL footprint was very close to the distal LA footprint of the ILFL; the two footprints were separated by a gap of a few millimetres. The mean length of the distal ISFL footprint was 11.3 mm and the mean width was 6.9 mm (Table 1). The mean length of the femoral neck was 26.7 mm and the F/FN ratio was 42.6% (Table 2).

## Discussion

This study provides a detailed quantitative description of the insertional footprints of the ILFL and ISFL and has two main findings. First, the relationship between the footprint of the ILFL and intertrochanteric line was determined using the F/I ratio. Second, the footprint of the ISFL located anterior to the femoral neck axis was described using the F/FN ratio. The F/I and F/FN ratios indicate the relative location of the insertional footprints between bony landmarks, which can be detected easily even under a narrow surgical view. Using these ratios, surgeons can reconstruct or preserve the capsular ligaments without causing unnecessary damage to the soft tissue. In this study, all assessments were performed using fresh frozen cadaver specimens. A fresh frozen cadaver is currently the closest model to reality and has advantages in terms of the quality of soft tissue, including minimal tissue changes with realistic hardness, colour, and pliability (Table 3) [18, 19]. Therefore, we considered the fresh frozen cadaver to be the most suitable model for our study.

**Table 3:** Characteristics of fresh frozen cadavers and formalin-fixed cadavers

	Advantages	Disadvantages
Fresh frozen	Flexible joints and tissue, minimal soft tissue change (realistic colour and hardness)	Risk of infection
Formalin-fixed	Minimal infection risk, good histological quality	Stiff joints and tissues, discoloration, unnatural texture

The importance of preserving the capsule in THA was highlighted by Fujishiro et al., who investigated the outcomes of reconstruction of the ILFL during THA to prevent recurrence of anterior dislocation and concluded that repair and correct tensioning of the ILFL could be the key to controlling anterior instability after THA [8]. Myers et al. and Matsuda et al. found that capsulectomy and capsulotomy during hip arthroscopy could cause iatrogenic hip instability and recommended that the ligaments of the hip capsule be repaired [5, 12]. In these reports, there is general agreement about the significance of preserving the ligaments in the hip capsule to maintain the defences of the hip against postoperative instability, including dislocation and subluxation.

Previous anatomical studies have described the ILFL in detail [9, 11, 13–16]. The present study also investigated the anatomical features of the ILFL, including its origin and size. The ILFL originated proximally from the base of the AIIS and inserted distally along the intertrochanteric line in the thicker peripheral portions (divided into the LA and MA) and in a thinner central portion between the LA and MA, which is fairly consistent with previous reports [13, 14]. However, the size of the LA and MA footprints in the present study were smaller than reported previously. The LA was found to have a mean footprint length of 23 mm (range, 17.3–27.8 mm; SD 3.5) and a width of 9.7 mm (range, 8.2–12.0 mm; SD 1.1) whereas Telleria et al. reported respective values of 38 mm (range, 26–58 mm; SD 9) and 11 mm (range, 6–16 mm; SD 3). Furthermore, in this study, the MA had a mean footprint length of 17.9 mm (range, 14.9–20.2 mm; SD 1.6) and width of 9.0 mm (range, 7.8–11.4 mm; SD 1.1) whereas the respective values reported by Telleria et al. were 31 mm (range, 21–38 mm; SD 6) and 20 mm (range, 12–29 mm; SD 6) [13]. The footprint sizes in their study were generally larger than in ours and may reflect differences in the race, sex, or physique of the specimens used in the two studies.

This study focused on the intertrochanteric line as a palpable osseous landmark for preserving the ILFL during surgery. It is possible to palpate the full length of the intertrochanteric line during THA using an anterior or anterolateral approach. Given that the distal footprints of both the MA and LA were attached to the intertrochanteric line, an understanding of the relationship between the distal ILFL footprint and the intertrochanteric line would be useful. This is the first report to propose the F/I ratio, defined as the ratio of the length of the ILFL footprint to the length of the intertrochanteric line, as a landmark for detecting the distal ILFL footprint during surgery. In this study, the mean F/I ratios for the MA and LA were 32.9 and 42.0%, respectively. Therefore, the distal MA footprint occupied the distal third of the intertrochanteric line and the distal LA footprint occupied the proximal 42% of this line. The lesser trochanter is also a palpable osseous landmark. Telleria et al. reported that the mean distance from the distal MA footprint to the lesser trochanter was 20 (range 15–27) mm [13]. The mean distance of 24.6 mm in the present study is slightly longer, but the range (19.9–28.5 mm) nearly overlaps. This distance might be helpful for detecting the distal MA footprint regardless of race or physique.

Some important reports have shown the anatomical features of the ILFL. Tsutsumi et al. investigated the capsular attachment of the anterosuperior aspect of the acetabulum, the proximal attachment site of the ILFL, based on osseous morphology and histological analyses. They found that the inferior edge of the AIIS was characterized by an osseous impression, a broad attachment width, and distributed fibrocartilage. These anatomical features were thought to be a response to mechanical stress and provide a new understanding of the importance of the proximal attachment of the ILFL for joint stability [15]. In a subsequent study, Tsutsumi et al. performed macroscopic and histological analyses that found the gluteus minimus tendon and deep aponeurosis of the iliopsoas to be continuous with the joint capsule [16]. Based on these new insights, they suggested that the ILFL could be regarded as not only a static stabilizer but also a dynamic stabilizer in the hip joint, which is able to transmit muscle power via the capsular complex. They also found that the gluteus minimus tendon could not be separated from the joint capsule for histological study. In the present study, we were able to observe the connection between the hip capsule and the aponeurosis of the muscles and we cut the base of this connection by detaching the insertion of the gluteus minimus. Although each footprint measurement in our study might include the connective tissue, the complex including the ILFL and gluteus minimus tendon itself should be functionally important. Therefore, our results will be useful in the clinical setting, and surgeons should understand this complex, including the muscle and capsule, to minimize any functional damage to the hip joint during surgery.

Our findings also indicate that the distal ISFL footprint was attached at the junction of the femoral neck and greater trochanter, which is located anterior to the femoral neck axis. This finding is similar to that in the report by Telleria et al. [13]. However, there are reports in major journals describing insertion of the ISFL in the posterior aspect of the femoral neck [7, 20]. Many surgeons may misunderstand the location of insertion of the ISFL and manage this ligament inappropriately, leading to serious complications, such as iatrogenic joint laxity, dislocation of the artificial joint, intraoperative fracture, or malalignment of the stem in THA [20]. The present study also evaluated the relationship between the anteroposterior femoral neck length at the base of the junction of the femoral neck and greater trochanter and the distal ISFL footprint. The distal ISFL footprint accounted for 42.6% of the front of the anteroposterior femoral neck length. There was no case in which the distal ISFL footprint was located behind the femoral neck axis. Although the ISFL reinforces the posterior portion of the hip capsule, the footprint of the distal ISFL was located relatively forward. There was a small gap between the footprint of the ISFL and the distal LA of the ILFL; however, this gap would not be clear enough to be identified during surgery. Therefore, careful incision is needed to preserve the ISFL during surgery.

There are several limitations to this study. First, the sample size was small and only Japanese cadavers were used. Therefore, it was not possible to identify any potential differences according to physique, sex, or race. However, there was no anatomical variation in the capsular ligaments in this series, and the SD for each measurement was very small. These data may be representative of the general population of Japan. Second, the joint position was not fixed when the measurements were obtained. All parameters in this study were measured when the ligament was in the maximally stretched position because of the ease of detection of the ligament area. However, it is unlikely that this would have influenced the results of the study because the size of the ligament footprint and the osseous structures did not change in this hip position. Third, none of the hip joints included in the study had osteoarthritis. Considering that the ligaments of the hip capsule can be thickened or shortened and that bony deformity or osteophytes can be observed in patients with osteoarthritis of the hip, the condition of the hip joint, including the capsular ligaments and bony structure, may be different during THA in the clinical setting. Finally, this study included only elderly cadaveric specimens (aged 69–96 years at the time of death). There is a possibility that the running and footprint of these ligaments may change with age.

## Conclusions

This study evaluated the detailed anatomy of the hip capsular ligaments and defined osseous landmarks that are useful for detecting the ILFL and ISFL during surgery. The F/I and F/FN ratios may help surgeons to manage the hip capsule intraoperatively even under a narrow surgical view. The findings of this study underscore the importance of understanding that the distal ISFL footprint is located relatively forward and very close to the distal LA footprint.

## Data Availability

The data that support the findings of this study are available from the corresponding author upon reasonable request.

## Abbreviations

THA: Total hip arthroplasty
ILFL: Iliofemoral ligament
LA: Lateral arm
MA: Medial arm
ISFL: Ischiofemoral ligament
SD: Standard deviation
